# Vessel wall MRI in moyamoya disease: arterial wall enhancement varies depending on age, arteries, and disease progression

**DOI:** 10.1007/s00330-023-10251-9

**Published:** 2023-10-05

**Authors:** Hiroshi Tagawa, Yasutaka Fushimi, Takeshi Funaki, Satoshi Nakajima, Akihiko Sakata, Sachi Okuchi, Takuya Hinoda, John Grinstead, Sinyeob Ahn, Yu Hidaka, Kazumichi Yoshida, Susumu Miyamoto, Yuji Nakamoto

**Affiliations:** 1 Department of Diagnostic Imaging and Nuclear Medicine, Graduate School of Medicine, Kyoto University, 54 Shogoin Kawahara-cho, Sakyo-ku, Kyoto, 606-8507 Japan; 2 Department of Neurosurgery, Graduate School of Medicine, Kyoto University, Kyoto, Japan; 3 Siemens Healthineers, Portland, OR USA; 4 Siemens Healthineers, San Francisco, CA USA; 5 Department of Biomedical Statistics and Bioinformatics, Graduate School of Medicine, Kyoto University, Kyoto, Japan

**Keywords:** Moyamoya disease, Magnetic resonance imaging, Adult, Child

## Abstract

**Objective:**

To investigate the relationship of followings for patients with moyamoya disease (MMD): arterial wall enhancement on vessel wall MRI (VW-MRI), cross-sectional area (CSA), time-of-flight MR angiography (MRA), age, locations from intracranial internal carotid artery (ICA) to proximal middle cerebral artery (MCA), disease progression, and transient ischemic attack (TIA).

**Methods:**

Patients who underwent VW-MRI between October 2018 and December 2020 were enrolled in this retrospective study. We measured arterial wall enhancement (enhancement ratio, ER) and CSA at five sections of ICA and MCA. Also, we scored MRA findings. Multiple linear regression (MLR) analysis was performed to explore the associations between ER, age, MRA score, CSA, history of TIA, and surgical revascularization.

**Results:**

We investigated 102 sides of 51 patients with MMD (35 women, 16 men, mean age 31 years ± 18 [standard deviation]). ER for MRA score 2 (signal discontinuity) was higher than ER for other scores in sections D (end of ICA) and E (proximal MCA) on MLR analysis. ER in section E was significantly higher in patients for MRA score 2 with TIA history than without. ER significantly increased as CSA increased in section E, which suggests ER becomes less in decreased CSA due to negative remodeling.

**Conclusion:**

Arterial wall enhancement in MMD varies by age, location, and disease progression. Arterial wall enhancement may be stronger in the progressive stage of MMD. Arterial wall enhancement increases with history of TIA at proximal MCA, which may indicate the progression of the disease.

**Clinical relevance statement:**

Arterial wall enhancement in moyamoya disease varies by age, location of arteries, and disease progression, and arterial wall enhancement may be used as an imaging biomarker of moyamoya disease.

**Key Points:**

- *It has not been clarified what arterial wall enhancement in moyamoya disease represents.*
- *Arterial wall enhancement in moyamoya disease varies by age, location of arteries, and disease progression.*
- *Arterial wall enhancement in moyamoya disease increases as the disease progresses.*

**Graphical abstract:**

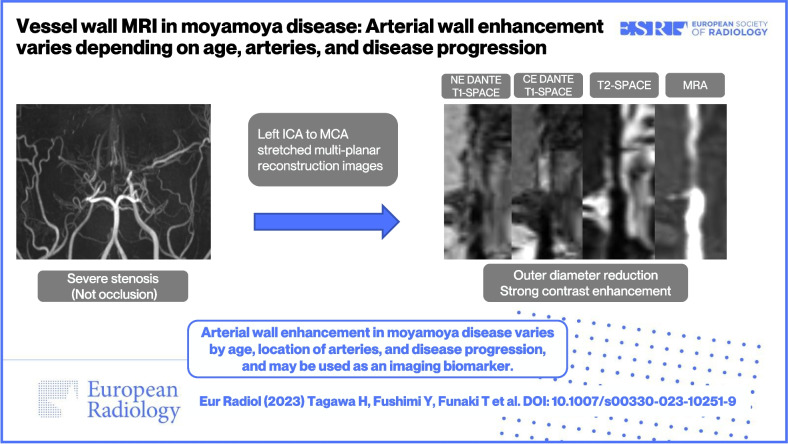

**Supplementary Information:**

The online version contains supplementary material available at 10.1007/s00330-023-10251-9.

## Introduction

Moyamoya disease (MMD) is characterized by progressive stenosis involving the terminal internal carotid artery (ICA) with small collateral vessels [1]. Although MMD is an idiopathic disease, accelerated vascular remodeling might underlie the pathological consequences of MMD [2], and the gene for ring finger protein (RNF) 213 has been identified as a susceptibility gene [3–5]. The incidence of MMD peaks in two age groups: children of approximately 5 years old and adults in their mid-40s [6, 7]. Diagnosis and staging of the disease are usually made based on conventional angiography focusing on the appearance, development, diminishment, and disappearance of moyamoya vessels [1]. Since the usefulness of MRI was reported, MRI has played a vital role in the treatment of MMD because of its lower invasiveness and high ability to detect cerebral infarction or hemorrhage [8, 9]. With the introduction of MR angiography (MRA), MR has mainly been used because the diagnostic capability for MMD is comparable to conventional angiography [10, 11]. The arterial shrinkage on 3D T2-weighted imaging is also helpful in differentiation from atherosclerotic disease [12]. Whereas the accumulation of the knowledge of imaging findings for MMD, there is no therapeutic option to stop the progression of MMD, and surgical revascularization is the main treatment. Angiography, nuclear medicine, MRA, and 3D T2-weighted imaging are excellent for assessing the current status of MMD and very helpful in determining the indications for surgical intervention [13]. However, these findings only reflect the result of vascular stenosis caused by MMD, and biomarkers that reflect the disease activities have not yet been developed. The emergence of biomarkers is expected to help in treating MMD and deciding surgical indications.

Vessel wall imaging (VWI) by MRI has recently come into use for evaluating larger vessels. Several studies have also reported the application of VWI for intracranial diseases, including MMD [14]. Although initial studies for a small number of cases reported a lack of or only weak contrast enhancement for vessel walls [15, 16], recent studies have shown a high frequency of ICA and middle cerebral artery (MCA) wall enhancement [17]. Another study revealed negative remodeling of the arteries in patients with MMD [18]. However, most recent studies have focused on differentiating MMD from atherosclerotic disease, and only a limited number have focused on the relationship between VWI and disease status [19]. Consequently, the clinical usefulness of VWI for MMD has yet to be established compared to MRA and 3D T2-weighted image.

We expected that if the association between VWI findings determined by enhancement ratio and radiographic progression is clarified, it could be used in clinical practice as an imaging biomarker of the disease activity, especially for the indication of surgical revascularization. For this purpose, we investigated the relationship between VWI findings, MRA findings determined by MRA scores, and cross-sectional area (CSA) in this study. The association with transient ischemic attack (TIA), a clinical indicator of the disease progression, was also examined.

## Methods

### Patients

**Fig. 1:**
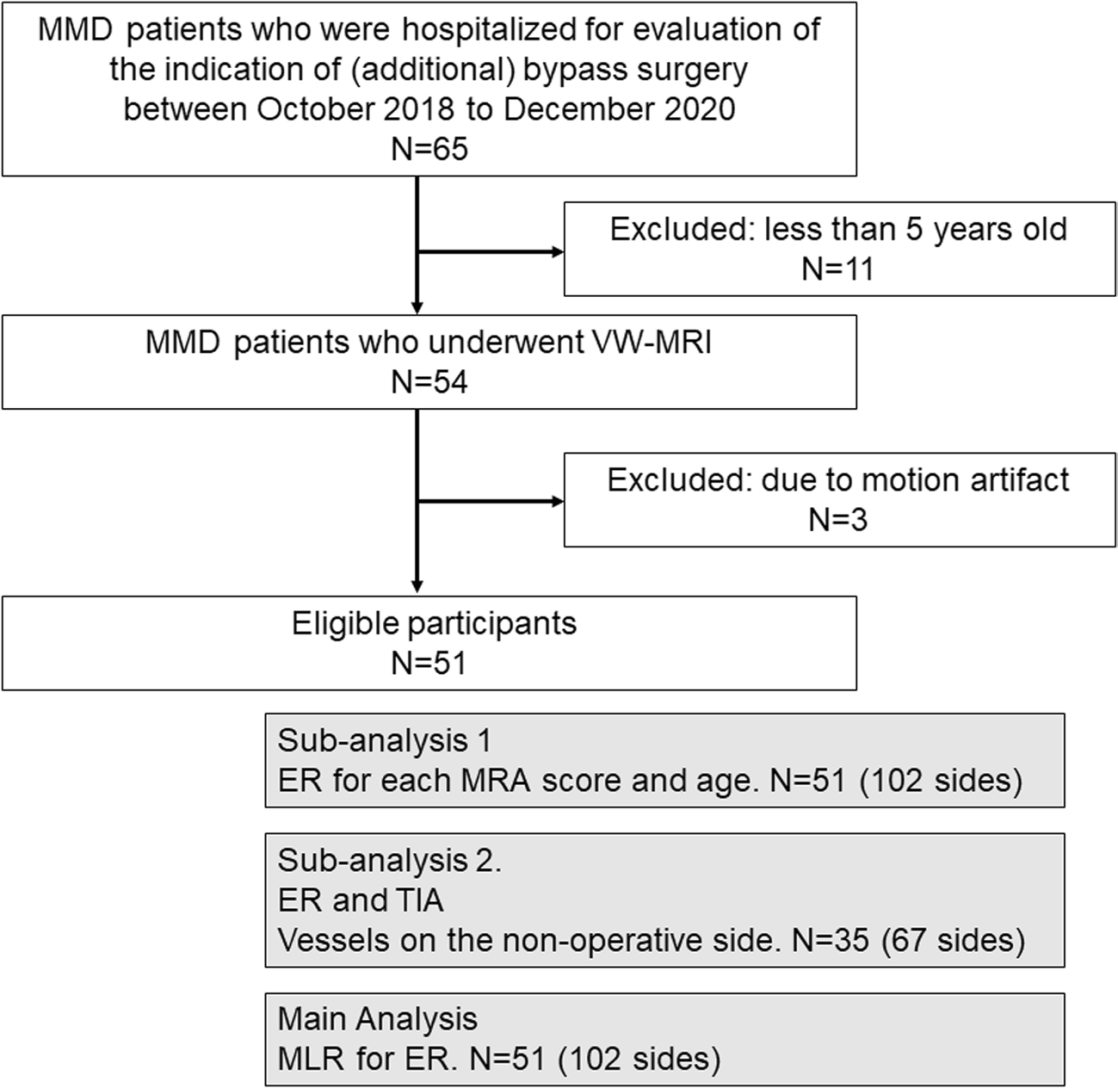
Flowchart of participants with MMD and subjects of analysis. MMD, moyamoya disease; MRA, magnetic resonance angiography; VWI, vessel wall imaging; CSA, cross-sectional area; TIA, transient ischemic attack; ER, enhancement ratio; MLR, multiple linear regression

This study was approved by the research ethics committee, and the need to obtain written informed consent was waived based on the retrospective design applied. Adult and pediatric patients with MMD who were hospitalized for evaluation of the indication of (additional) bypass surgery between October 2018 and December 2020 and underwent VWI on 3-T MRI were enrolled (Fig. 1). All participants were Japanese and were diagnosed with MMD by conventional angiography. Exclusion criteria were patients less than 5 years old, patients with asthma or allergy to gadolinium-based contrast media, patients with renal insufficiency, and patients with motion artifact during MR scan. Pediatric patients who could not remain at rest were sedated during an MR scan. Patients less than 5 years were excluded because the usefulness of CE VWI findings had not been established in MMD. In addition, some of these infants were in unstable condition, and the risks of adverse effects of gadolinium-based contrast agent and sedation should be avoided in terms of patient management. We reviewed the history of surgical revascularization and TIA within the 3 months preceding the MRI scan date. If the patient experienced a unilateral transient episode of neurological dysfunction within 3 months, the cerebral hemisphere responsible for the symptoms was defined as having a history of TIA. Because the progression of MMD often differs between the left and right sides [20], both sides of a patient were treated independently.

### MRI protocol

Additional preparation pulses, delay alternating with nutation for tailored excitation (DANTE) pulses, were used for VWI to achieve good black-blood effects as well as cerebrospinal fluid (CSF) signal suppression in this study, as have been used for other studies [21–26]. MRI, including non-contrast-enhanced (NE) and contrast-enhanced (CE) DANTE T1-sampling perfection with application optimized contrast using different flip angle evolution (SPACE), was conducted using a 3-T MRI unit (MAGNETOM Prisma or Skyra; Siemens Healthineers) with a 32-channel head coil or 64-channel head/neck coil. Gadolinium-based contrast agent (Gadobutrol, 0.1 mmol/kg, Bayer AG) was administered intravenously. Imaging parameters of DANTE T1-SPACE, NE Time-of-Flight (TOF)-MRA, and NE 3D T2-SPACE are shown in the Supplementary Annex.

### MRA score

**Fig. 2:**
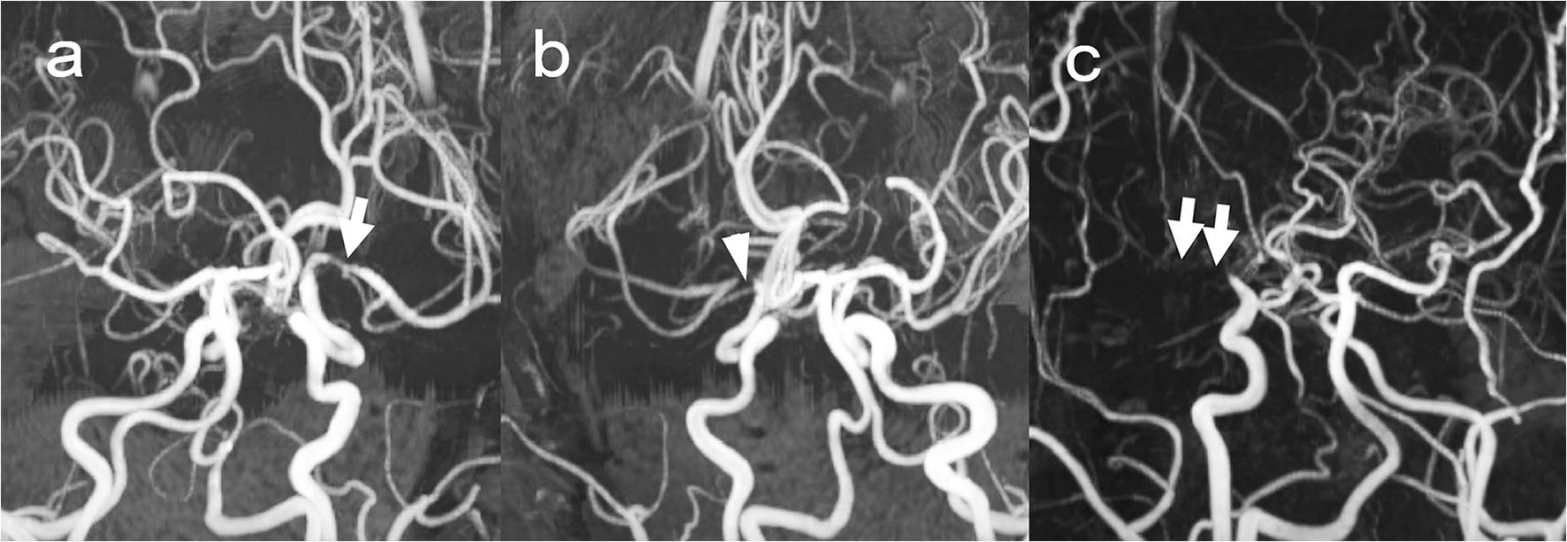
Examples of MRA score on MIP images of TOF-MRA. **a** MRA score 1: stenosis (arrow); **b** MRA score 2: signal discontinuity (arrowhead); **c** MRA score 3: invisible (arrows). MIP, maximum intensity projection; TOF-MRA, time-of-flight MR angiography

MRA score has been developed to assess MMD grade [10]. Two board-certificated radiologists (S.O. and H.T., with 14 and 9 years of experience in neuroradiology, respectively) determined MRA scores on maximum intensity projection (MIP) of TOF-MRA using a 4-point scale (0: normal; 1: stenosis; 2: signal discontinuity; 3: invisible) from the intracranial ICA to the proximal third of the horizontal segment of the MCA (Fig. 2). The final score was determined as a consensus decision if the score did not match between readers. We assumed MRA score 2 as active phase of radiographic progression when the risk of ischemia increases markedly and surgical indications are considered.

### Enhancement ratio (ER) and CSA

**Fig. 3:**
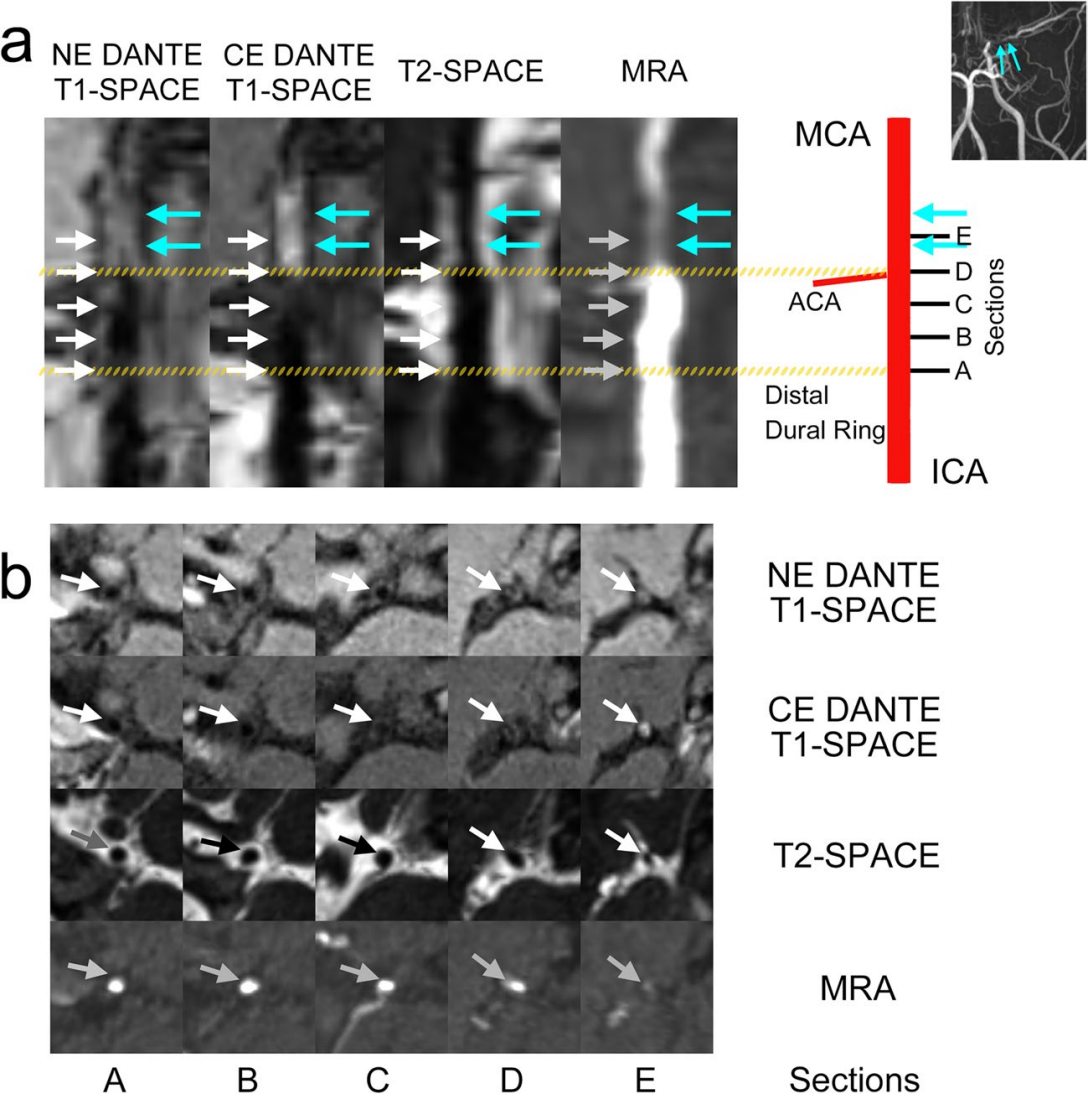
Case 1. Left-side ICA-MCA of an 18-year-old man. On the MIP image, the signal of the proximal MCA was discontinuous (MRA score 2). **a** Stretched MPR images of NE DANTE T1-SPACE, CE DANTE T1-SPACE, T2-SPACE, and MRA and schema of the 5 observed sections. Note that distances between each section are equal. Section A, start of the intracranial ICA (across the distal dural ring); section B, proximal intracranial ICA; section C, distal intracranial ICA; section D, end of the ICA; and section E, proximal MCA. Stenosis is seen in the proximal portion of the MCA (cyan arrows), and T2-SPACE also shows a reduction in outer diameter. As compared to NE DANTE T1-SPACE, CE DANTE T1-SPACE shows strong contrast enhancement (cyan arrows). **b** On axial stretched MPR images, the ICA and MCA are indicated by white, gray, and black arrows. CE DANTE T1-SPACE shows enhancement from sections D to E (end of ICA to proximal MCA), T2-SPACE shows mild narrowing of the artery, and MRA shows decreased inflow

First, we performed registration of NE DANTE T1-SPACE images and T2-SPACE images to CE DANTE T1-SPACE images with 3D Slicer software on a workstation (https://www.slicer.org/) [27]. Next, stretched multi-planar reconstruction (MPR) images along the ICA to MCA were created using a curved planar reformat plugin (SlicerSandbox) for 3D Slicer software. We then set five equally spaced sections for observation of the intracranial ICA and proximal MCA: section A, start of the intracranial ICA (across the distal dural ring); section B, proximal intracranial ICA; section C, distal intracranial ICA; section D, end of the ICA; and section E, proximal MCA (Fig. 3). We chose these five sections to focus on the disease status of MMD from intracranial ICA to proximal MCA, where angiographic abnormalities and pathological findings are most likely observed.

Signal intensity was measured by importing images from 3D Slicer into ImageJ (https://imagej.nih.gov/ij/index.html) [28]. Maximal signal intensities of the arteries were measured at each section on pre- and post-contrast DANTE-T1-SPACE (VW_NE_ and VW_CE_, respectively) by one board-certified radiologist (H.T., 9 years of experience in neuroradiology). The region of interest (ROI) was manually set along the outer contour of the artery, including the lumen (Supplementary Figure 4). Maximum signal intensity instead of the mean signal intensity in the ROI was chosen because with the progression of MMD, the arteries become very small, and the inner contour becomes obscured, making it very difficult to determine the exact ROI of the vessel wall. T2-SPACE images were also referred to when the arteries were very small in diameter and difficult to be recognized with DANTE T1-SPACE. As control values, mean mid-brain intensity (MB_NE_ and MB_CE_) was also measured, then the ER was calculated as below.

$$\mathrm{ER}=\frac{{~}^{{\mathrm{VW}}_{\mathrm{CE}}}\!\left/ \!{~}_{\mathrm{V}{\mathrm{W}}_{\mathrm{NE}}}\right.}{{~}^{{\mathrm{MB}}_{\mathrm{CE}}}\!\left/ \!{~}_{{\mathrm{MB}}_{\mathrm{NE}}}\right.}$$

CSA was measured in each section on T2-SPACE images imported from 3D-slicer using ImageJ. In each section, the ROI was manually set along the outer contour of the arterial wall, and the ROI’s area was defined as CSA (Supplementary Figure 4).

### Sub-analysis 1: ER for each MRA score and age

We compared ER between patients with different MRA scores using the Kruskal-Wallis test and Mann-Whitney *U* tests with Bonferroni correction. The comparisons were performed for all patients, the pediatric group (age < 20 years), and the adult group (age ≥ 20 years). Also, we compared ER between the pediatric group (age < 20 years) and the adult group (age ≥ 20 years) for each MRA score with Mann-Whitney *U* tests.

### Sub-analysis 2: ER and TIA

Since surgical revascularization is performed to reduce the risk of ischemia associated with stenosis of the corresponding native arteries, it is difficult to evaluate the relationship between VWI findings and TIA after bypass surgery. Therefore, arteries that had undergone bypass surgery were excluded from sub-analysis 2. We compared differences in ER for each MRA score between arteries with and without a history of TIA using Mann-Whitney *U* tests.

### Main analysis: multiple linear regression (MLR) for ER

We used ER as the dependent variable in anticipation of its potential use as a biomarker. CSA, MRA score, age, history of TIA, and history of bypass surgery were independent variables. For MRA score, we used indicator variables (scores 1, 2, and 3), grouping MRA scores 0 and 1 together as score 1 due to the small number of cases and optimizing the number of variables for MLR.

### Statistical analysis

We used JMP Pro version 16.1 software (SAS Institute). Values of *p* < 0.05 were considered statistically significant. For sub-analysis 1 with Bonferroni correction, *p* values < 0.0083 were considered significant.

## Results

### Patients

Sixty-five patients were enrolled in this study. Eleven patients were excluded due to being less than 5 years old, and three patients were excluded due to severe motion artifacts, so 102 sides of 51 patients (16 men, 35 women; median age, 31 years; range, 5–66 years; 21 patients were < 20 years old) were included finally (Fig. 1). Seventeen patients had a history of TIA within the preceding 3 months (17 sides), and 26 patients had undergone bypass surgery (35 sides). The demographic characteristics of patients are shown in Table 1. Representative cases are shown in Fig. 3 and Supplementary Figures 1, 2, and 3.

**Table 1:** Demographics and clinical data of patients

Patients	
Female (*n*) (age < 20)	35 (12)
Male (*n*) (age < 20)	16 (9)
Age (years) (mean ± SD, range)	31 ± 18 (5–66)
History of bypass surgery (side) (age < 20)	35 (20)
History of TIA without surgery (side) (age < 20)	12 (6)
MRA score (side) (age < 20)	
Score 0	10 (5)
Score 1	8 (5)
Score 2	25 (16)
Score 3	59 (16)

The number of patients under 20 years old is shown in parentheses. Note that the 4-point scale for MRA score represents the following: 0, normal; 1, stenosis; 2, signal discontinuity; and 3, invisible. *TIA*, transient ischemic attack

### MRA score

MRA scores from different readers were in almost perfect agreement, with concordance for 94 of 102 scores (Cohen’s *κ* = 0.87). The number of sides with each MRA score is shown in Table 1.

### ER and CSA

**Fig. 4:**
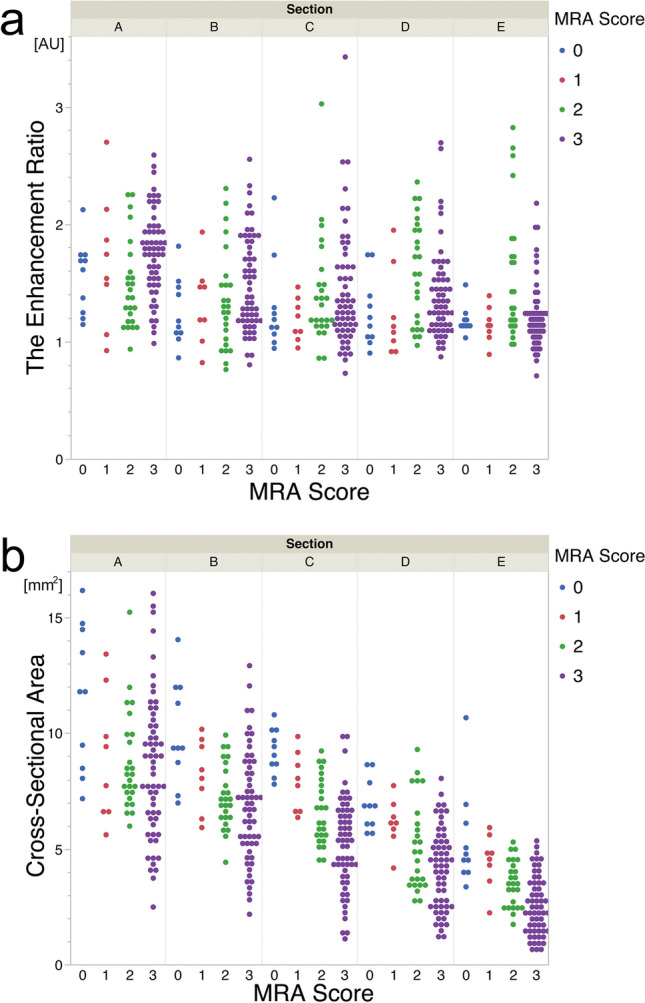
**a** The plot of ER for each MRA score in each section; ER of MRA score 2 appears larger in sections D and E, suggesting the possibility of a non-proportional relationship between enhancement ratio and MRA score. **b** The plot of CSA for each MRA score in each section; CSA appears to decrease as MRA score increases. MRA score 0, normal (blue); MRA score 1, stenosis (red); MRA score 2, signal discontinuity (green); and MRA score 3, invisible (purple). Five observed sections of the intracranial ICA and proximal MCA: section A, start of intracranial ICA (across the distal dural ring); section B, proximal intracranial ICA; section C, distal intracranial ICA; section D, end of the ICA; and section E, proximal MCA. ER, enhancement ratio. CSA, cross-sectional area

Mean ER and CSA for each MRA score are shown in Table 2. ER and CSA for each MRA score in each section are plotted in Fig. 4. ER was highest for MRA score 3 in sections A and B, whereas it was highest for score 2 in sections D and E. CSA decreased as MRA score increased.

**Table 2:** ER and CSA for each MRA score in each section

ER (AU)						
		Section A	Section B	Section C	Section D	Section E
MRA score	0	1.55 ± 0.31	1.25 ± 0.29	1.29 ± 0.40	1.25 ± 0.30	1.18 ± 0.12
	1	1.68 ± 0.57	1.32 ± 0.36	1.19 ± 0.18	1.24 ± 0.38	1.14 ± 0.15
	2	1.48 ± 0.37	1.34 ± 0.43	1.41 ± 0.46	1.60 ± 0.43	1.55 ± 0.55
	3	1.76 ± 0.36	1.48 ± 0.40	1.41 ± 0.50	1.39 ± 0.38	1.21 ± 0.28
CSA (mm^2^)						
		Section A	Section B	Section C	Section D	Section E
MRA score	0	11.58 ± 3.15	10.06 ± 2.24	9.26 ± 0.97	6.94 ± 1.12	5.39 ± 2.13
	1	8.96 ± 2.82	8.22 ± 1.56	7.86 ± 1.33	6.11 ± 1.03	4.48 ± 1.16
	2	8.71 ± 2.14	7.27 ± 1.38	6.56 ± 1.39	5.11 ± 1.93	3.49 ± 0.98
	3	8.48 ± 3.07	6.77 ± 2.39	5.12 ± 1.98	4.10 ± 1.73	2.48 ± 1.24

ER (AU) and CSA (mm^2^) (mean ± standard deviation) of each MRA score in each section. Note that the 4-point scale for MRA score represents the following: 0, normal; 1, stenosis; 2, signal discontinuity; and 3, invisible. Five observed sections of the ICA-MCA represent the start of intracranial ICA (section A), proximal intracranial ICA (section B), distal intracranial ICA (section C), end of the ICA (section D), and proximal MCA (section E). *ER*, enhancement ratio; *AU*, arbitrary unit; *CSA*, cross-sectional area. *ICA*, internal carotid artery; *MCA*, middle cerebral artery

### Sub-analysis 1: ER for each MRA score and age

The Kruskal-Wallis test for all patients showed a significant difference in ER per MRA score in sections A, D, and E (*p* = 0.01, 0.02, and 0.03, respectively) (Table 3). Results of the Mann-Whitney *U* test are shown in Supplementary Table 1. With Bonferroni correction for multiple-comparison, ER for MRA score 2 was significantly higher than ER for MRA score 3 in section E among all patients (*p* = 0.006) (Supplementary Table 1).

**Table 3:** Comparison of ER between patients with different MRA scores: Kruskal-Wallis test

	Section A	Section B	Section C	Section D	Section E
All patients (102 sides)	0.01*	0.15	0.50	0.02*	0.03*
Adult patients (60 sides)	0.32	0.25	0.95	0.38	0.54
Pediatric patients (42 sides)	0.04*	0.69	0.50	0.06	0.02*

Kruskal-Wallis test was performed to compare ER for each MRA score in each section. *p* values for each section among all patients, among adult patients, and among pediatric patients are shown. Note that each subject with MRA score (0–3) is included. *p < 0.05

Section A, the start of the intracranial ICA (across the distal dural ring); section B, proximal intracranial ICA; section C, distal intracranial ICA; section D, end of the ICA; section E, proximal MCA; *ER*, enhancement ratio

The results of analysis comparing ER and MRA score among pediatric and adult patients are shown in Table 3 (Kruskal-Wallis test), Supplementary Tables 2 and 3 (Mann-Whitney *U* test). Comparison of ER between adult and pediatric patients for the same MRA score is shown in Table 4.

**Table 4:** ER of pediatric and adult patients for the same MRA score: Mann-Whitney *U* test

**MRA score 2, *n*** **= 25 (pediatric patients, 16; adult patients, 9)**						
Section	Age	Mean	SD	Mean rank	*Z*	*p* value
A	Pediatric	1.34	0.30	10.44	−2.29	0.02*
	Adult	1.73	0.39	17.56		
B	Pediatric	1.20	0.37	10.31	−2.41	0.02*
	Adult	1.61	0.41	17.78		
C	Pediatric	1.40	0.52	12.31	−0.59	0.55
	Adult	1.44	0.36	14.22		
D	Pediatric	1.63	0.43	13.63	0.54	0.59
	Adult	1.56	0.46	11.89		
E	Pediatric	1.59	0.50	14.44	1.27	0.20
	Adult	1.46	0.66	10.44		
**MRA score 3,** ***n*** **= 59 (pediatric patients, 16; adult patients, 43)**						
Section	Age	Mean	SD	Mean rank	*Z*	*p* value
A	Pediatric	1.63	0.28	24.00	−1.63	0.10
	Adult	1.81	0.38	32.23		
B	Pediatric	1.33	0.37	22.94	−1.92	0.06
	Adult	1.54	0.40	32.63		
C	Pediatric	1.27	0.47	23.63	−1.73	0.08
	Adult	1.46	0.50	32.37		
D	Pediatric	1.38	0.55	25.44	−1.24	0.22
	Adult	1.39	0.31	31.70		
E	Pediatric	1.18	0.29	26.44	−0.96	0.34
	Adult	1.22	0.28	31.33		

Note that MRA scores 0 and 1 were not compared due to the lack of number in adults and pediatrics. The asterisks (*) represent statistical significance (*p* values < 0.05)

Section A, the start of the intracranial ICA (across the distal dural ring); section B, proximal intracranial ICA; section C, distal intracranial ICA; section D, end of the ICA; section E, proximal MCA; *ER*, enhancement ratio

Among pediatric patients, significant differences in ER per MRA score were seen in sections A and E (*p* = 0.04 and 0.02, respectively) (Table 3). Conversely, no significant difference was observed among adult patients. Using the Mann-Whitney *U* test with Bonferroni correction, a significant difference in ER was observed between MRA scores 2 and 3 in sections A and E among pediatric patients (*p* = 0.004 and 0.007, respectively) (Supplementary Table 3).

For MRA score 2, ER was significantly higher in adult patients than in pediatric patients in sections A and B (*p* = 0.02 and 0.02, respectively) (Table 4).

### Sub-analysis 2: ER and TIA

Vessels on the non-operative side (67 sides) were analyzed. The ER of MRA score 2 was significantly higher in section E (proximal MCA) for patients with a history of TIA than for patients without a history of TIA (*p* = 0.02), while no significant difference was observed in section D (end of the ICA) (*p* = 0.48) (Supplementary Table 4). No significant differences in ER were seen for patients with MRA score 3.

### Main analysis: MLR for ER

The results of MLR analysis are shown in Table 5. ER tended to increase as CSA increased in section E (estimated parameter *B* = 0.065, 95% CI [0.009, 0.121], *p* = 0.02). Also, in section E, ER tended to be higher in the group with history of TIA (*B* = 0.117, 95% CI [0.016, 0.218], *p* = 0.02). In section B, ER has positive relation to age (*B* = 0.005, 95% CI [0.0004, 0.010], *p* = 0.03). MLR analysis did not find a significant relationship between ER and history of bypass surgery.

**Table 5:** The estimated parameter and confidence interval of each section derived from MLR analysis predicting ER

	Section A				Section B				Section C				Section D				Section E			
	*B*	95% CI		*p* value	*B*	95% CI		*p* value	*B*	95% CI		*p* value	*B*	95% CI		*p* value	*B*	95% CI		*p* value
		Lower	Upper			Lower	Upper			Lower	Upper			Lower	Upper			Lower	Upper	
Age	0.003	−0.001	0.008	0.14	0.005	0.0004	0.010	0.03*	0.001	−0.005	0.007	0.76	0.000	−0.005	0.005	0.94	−0.004	−0.008	0.0004	0.08
Surgery																				
(−)	Reference				Reference				Reference				Reference				Reference			
(+)	−0.006	−0.096	0.083	0.89	−0.034	−0.123	0.054	0.44	−0.098	−0.206	0.010	0.08	−0.022	−0.114	0.070	0.64	−0.025	−0.104	0.054	0.53
TIA																				
(−)	Reference				Reference				Reference				Reference				Reference			
(+)	−0.061	−0.173	0.051	0.28	−0.051	−0.165	0.064	0.38	0.053	−0.086	0.192	0.45	−0.041	−0.160	0.077	0.49	0.117	0.016	0.218	0.02*
CSA	0.008	−0.020	0.035	0.58	−0.033	−0.070	0.003	0.07	−0.010	−0.067	0.048	0.74	−0.011	−0.060	0.039	0.67	0.065	0.009	0.121	0.02*
MRA score																				
MRA-1	−0.020	−0.164	0.124	0.79	−0.042	−0.192	0.109	0.58	−0.100	−0.300	0.101	0.33	−0.170	−0.331	−0.009	0.04*	−0.219	−0.366	−0.071	0.004*
MRA-2	−0.106	−0.231	0.018	0.10	−0.012	−0.138	0.113	0.84	0.042	−0.110	0.193	0.59	0.204	0.074	0.334	0.003*	0.207	0.096	0.319	< 0.001*
MRA-3	Reference				Reference				Reference				Reference				Reference			

Section A, the start of the intracranial ICA (across the distal dural ring); section B, proximal intracranial ICA; section C, distal intracranial ICA; section D, end of the ICA; section E, proximal MCA; *ER*, enhancement ratio; *TIA*, history of TIA within the preceding 3 months; *Surgery*, history of bypass surgery; *CSA*, cross-sectional area; *MRA 1*, MRA scores 0 and 1; *MRA 2*, MRA score 2; *MLR*, multiple linear regression; *B*, estimated parameter; *CI*, confidence interval. For indicator variables of MRA, the reference category is MRA score 3. The asterisks (*) represent statistical significance (*p* values < 0.05)

Toward the periphery, the ER of MRA scores 0 and 1 tended to be smaller than that of MRA score 3 (estimated parameter *B* = −0.020 [section A], −0.042 [section B], −0.100 [section C], −0.170 [section D], −0.219 [section E]; statistically significance was observed in sections D [*p* = 0.04] and E [*p* = 0.004]). In the proximal region (sections A and B), the ER of MRA score 2 was lower than that of MRA score 3, whereas in the distal region (sections C, D, and E), the ER of MRA score 2 was higher than that of MRA score 3 (*B* = −0.106 [section A], −0.012 [section B], 0.042 [section C], 0.204 [section D], 0.207 [section E]; statistically significance was observed in sections D [*p* = 0.003] and E [*p* < 0.001]). Thus, ER for MRA score 2 was higher than ER for other scores in sections D and E.

## Discussion

This study investigated the relationship between arterial wall enhancement (as represented by ER), CSA, a 4-point-scale MRA score, age, and the history of TIA within 3 months and surgical revascularization in MMD patients. MLR analysis demonstrated that ER was higher with MRA score 2 (signal discontinuity) than with MRA score 3 in sections D (end of the ICA) and E (proximal MCA). In section E, there was a positive correlation between ER and CSA, representing that lower ER was observed in patients with smaller CSA. A positive correlation between ER and history of TIA was shown in section E; also, sub-analysis revealed ER with MRA score 2 was significantly stronger in patients with history of TIA within the preceding 3 months (*p* = 0.02) than without the history in section E. There were following differences of VWI findings between adult and pediatric patients: In the proximal portion of intracranial ICA (sections A and B), ER for MRA score 2 of adult patients was higher than that of pediatrics (*p* = 0.02 and 0.02, respectively). There was no apparent correlation between ER and history of surgical revascularization.

Arterial wall enhancement of MRA score 2 tended to be higher in sections D and E in this study (Table 5, Supplementary Table 1), although a previous study found no significant difference in the frequency of arterial wall enhancement in the MMD stage (Suzuki grade) [17]. We evaluated the intensity of contrast enhancement using the ER, and the good intravascular signal suppression and high resolution provided by DANTE-T1-SPACE may have contributed to these results [21–26]. Yang et al noted that the natural history of MMD is challenging to evaluate from contrast enhancement [29], but our results (including MLR analysis) suggest artery wall enhancement at the end of the ICA and proximal MCA reflects the disease progression of MMD.

This study also showed a negative correlation between MRA score and vascular CSA in section E (proximal MCA) (Table 2, Fig. 4), consistent with previous reports of negative vascular remodeling in MMD [18, 30, 31]. The positive correlation between ER and CSA in section E suggests that decreased CSA due to disease progression (negative remodeling) reduced arterial wall enhancement, and less decreased CSA in the active stage is associated with high arterial wall enhancement.

Pediatric patients in addition to adult patients were included in this study, and the relationship between age and ER was investigated, whereas most previous reports of VWI in MMD have focused on patients over 16 or 18 years old [17, 19, 32]. MMD has a bimodal distribution around 5 years old and in the 40s [6, 33, 34], so disease progression may differ between pediatric and adult patients. In our study, pediatric patients tended to display greater differences in arterial wall enhancement among MRA scores in sections A and E compared to adult patients (Table 3, Supplementary Table 3). This result may be related to differences in the disease progression of MMD between pediatric and adult patients. However, further investigation, including a larger number of pediatric and adult patients, is required.

The relationship between ER and history of TIA was assessed in this study, and contrast enhancement in section E was significantly stronger in patients with MRA score 2 and a history of TIA within the preceding 3 months (Supplementary Table 4). MLR similarly showed a larger ER for section E with a history of TIA (Table 5). Roder et al also reported that patients with new symptoms had substantial contrast enhancement of the vessel wall, which tended to diminish thereafter [19]. No significant difference in ER was found in section D in this study, probably because artery wall enhancement in patients (particularly those with MRA score 2) was strong regardless of the history of TIA.

ER was higher at the start of intracranial ICA (section A) and proximal intracranial ICA (section B), which was attributed to the contrast enhancement associated with factors other than MMD, such as dura mater, vasa vasorum, and venous plexus. In addition, ER in the proximal ICA was significantly higher in adult patients than in pediatric patients, which may be associated with the development of vasa vasorum with aging [28, 29]. MLR analysis also supported that age affects arterial wall enhancement in the proximal intracranial ICA, so this may represent a pitfall in the interpretation of findings from VW-MRI. However, in sub-analysis 1, ER was significantly higher for section A in all patients and in pediatric patients with MRA score 3 than the patients with MRA score 2 (Supplementary Table 1 and 3), suggesting that MMD may also affect the arterial wall enhancement in the proximal intracranial ICA.

The major limitation of this retrospective study was that only a single time point was investigated, and changes over time are unknown. We assumed MRA score 2 as “active phase of radiographic progression,” but it was unclear whether the stenosis would progress in the future or whether the contrast enhancement of the vessels made a difference in the rate at which the stenosis progressed. Few multi-time point studies have been reported to date [19]. Since MMD is a chronic progressive disease, we think that MRA findings can estimate the degree of disease progression, but further studies are required. Another limitation was that histopathological findings for areas showing arterial wall enhancement were not confirmed. Although thickening of the arterial intima is known to occur mainly in the terminal portion of ICA and the circle of Willis [12], it remains unclear what precisely the arterial wall enhancement reflects. Reports of pathologic findings in MMD are limited; one biopsy report demonstrated that the wall of MCA specimens showed intimal thickening as well as media thinning, and elongation, fragmentation, and disappearance of internal elastic lamina [21]. These findings have also been reported in autopsy patients [22, 23]. Additionally, inflammatory cell infiltration was generally absent, and mural thrombi were often seen in the stenotic lesion [23]. Intracranial arterial walls may show contrast enhancement due to vasa vasorum, which can be problematic in VWI interpretation [15]. Intracranial vasa vasorum is generally absent in children and becomes apparent with age [24]. Vascular stenosis may cause the development of vasa vasorum in MMD, but the development of vasa vasorum in MMD patients has not been elucidated. Further accumulation of knowledge on VW-MRI will contribute to the understanding of MMD pathophysiology and the enhancement of the vessel wall. In addition, because it is difficult to accurately circumscribe the ROI of the vessel wall in patients with advanced MMD, and in this study, the signal intensity of the vessels, including the lumen, was measured. Therefore, the possibility that intravascular findings (e.g., thrombus or flow artifact) were included in the contrast enhancement could not be excluded. Next, compared to MRA scores 2 and 3, the number of patients with scores 0 and 1 is small. Therefore, statistical analyses that are related to the early stages of MMD may have insufficient power. Last, we treated the left and right vessels as independent, but it is not guaranteed that they are completely independent, as patient-specific factors may influence the ER and other findings.

In conclusion, arterial wall enhancement in MMD varies by age, location of arteries, and disease progression. The arterial wall enhancement of the distal ICA and the proximal MCA may be stronger in the progressive stage of MMD. A correlation was identified between arterial wall enhancement and history of TIA at the proximal MCA, which may indicate the activity of MMD as well as the risk of cerebral ischemia. The correlation between arterial wall enhancement and the progression of MMD may suggest its potential as an imaging biomarker.

### Supplementary information

ESM 1 (PDF 375 kb)

## Abbreviations

CAIPIRINHA: Controlled aliasing in parallel imaging results in higher acceleration
CE: Contrast-enhanced
CI: Confidence interval
CSA: Cross-sectional area
CSF: Cerebrospinal fluid
DANTE: Delay alternating with nutation for tailored excitation
ER: Enhancement ratio
ICA: Internal carotid artery
MCA: Middle cerebral artery
MIP: Maximum intensity projection
MLR: Multiple linear regression
MMD: Moyamoya disease
MPR: Multi-planar reconstruction
MRA: MR angiography
NE: Non-contrast enhanced
RNF: Ring finger protein
SPACE: Sampling perfection with application optimized contrast using different flip angle evolution
TIA: Transient ischemic attack
TOF: Time-of-flight
VWI: Vessel wall imaging

## Declarations

### Guarantor

The scientific guarantor of this publication is Yuji Nakamoto.

### Statistics and Biometry

We included one statistician as a co-author.

Dr. Yu Hidaka, Department of Biomedical Statistics and Bioinformatics, Graduate School of Medicine, Kyoto University, Kyoto, 606-8507 Japan.

### Study subjects or cohorts overlap

There is no overlap in this study.

### Methodology

- • Retrospective
- • cross sectional study
- • performed at one institution
