# Supplementary material for: Vessel wall MRI in moyamoya disease: arterial wall enhancement varies depending on age, arteries, and disease progression
Source: Eur Radiol. 2023 Oct 5;34(4):2183–94. doi: 10.1007/s00330-023-10251-9 (PMC11322227; doi:10.1007/s00330-023-10251-9)
Supplement: Supplementary file 1 — (PDF 375 kb) [file 330_2023_10251_MOESM1_ESM.pdf]

**Vessel wall MRI in moyamoya disease: Arterial wall enhancement varies depending on age, arteries, and disease progression**

**Electronic Supplementary Material**

**Supplementary Annex**

Imaging parameters were as follows.

DANTE T1-SPACE: TR 1000 ms; TE, 11 ms; variable flip angle; echo train length, 60; field of view, 180 × 180 mm; resolution, 0.56 × 0.56 mm; matrix size, 320 × 320; slice thickness, 0.56 mm (isotropic voxels, 0.56 mm); number of slices, 256; acceleration with controlled aliasing in parallel imaging results in higher acceleration (CAIPIRINHA), 4×; fat suppression; whole-brain coverage; and acquisition time, 5 min 44 s. Parameters for the DANTE pulse were as follows: flip angle, 10°; radiofrequency (RF) duration, 0.08 ms; number of pulses, 148; total pulse duration, 167.24 ms; spoiler gradient area, 18 mT/m×ms.

NE Time-of-Flight (TOF)-MRA coverage of almost the entire brain was obtained by adjusting the number of slabs: axial acquisition; TR, 21 ms; TE, 3.7 ms; flip angle, 20°; echo train length, 68; field of view, 168–187 × 220 mm; matrix, 328 × 384; slice thickness, 0.7 mm; number of slices, 156–224; number of slabs, 4–5; and acquisition time, 5 min 50 s to 7 min 18 s.

NE 3D T2-SPACE was performed to visualize the outer wall of the ICA and MCA in the cisterns: axial acquisition; TR, 1100 ms; TE, 199 ms; flip angle, 140°; echo train length,

68; field of view, 200 × 200 mm; matrix, 320 × 320; resolution, 0.63 × 0.63 mm; slice thickness, 0.6 mm; number of slices, 80; and acquisition time, 4 min 2 s.

## Supplementary Tables

**Supplementary Table 1.** Comparison of enhancement ratio between MRA scores in each section for all patients.

Section A — Subtraction

| MRA Score | | Mean | SD | Z | p-value |
|---|---|---|---|---|---|
| 1 | 0 | 1.24 | 2.53 | 0.49 | 0.63 |
| 2 | 0 | -3.15 | 3.83 | -0.82 | 0.41 |
| 2 | 1 | -3.55 | 3.93 | -0.90 | 0.37 |
| 3 | 0 | 12.92 | 6.86 | 1.88 | 0.06 |
| 3 | 1 | 5.18 | 7.34 | 0.71 | 0.48 |
| 3 | 2 | 18.28 | 5.82 | 3.14 | 0.002* |

Section B

| MRA Score | | Mean | SD | Z | p-value |
|---|---|---|---|---|---|
| 1 | 0 | 1.69 | 2.53 | 0.67 | 0.51 |
| 2 | 0 | 1.61 | 3.83 | 0.42 | 0.67 |
| 2 | 1 | -0.083 | 3.93 | -0.021 | 0.98 |
| 3 | 0 | 13.27 | 6.86 | 1.93 | 0.05 |
| 3 | 1 | 7.45 | 7.34 | 1.02 | 0.31 |
| 3 | 2 | 8.83 | 5.82 | 1.52 | 0.13 |

Section C

| MRA Score | | Mean | SD | Z | p-value |
|---|---|---|---|---|---|
| 1 | 0 | -0.11 | 2.53 | -0.044 | 0.96 |
| 2 | 0 | 3.99 | 3.83 | 1.04 | 0.30 |
| 2 | 1 | 5.53 | 3.93 | 1.41 | 0.16 |
| 3 | 0 | 5.67 | 6.86 | 0.83 | 0.41 |
| 3 | 1 | 7.74 | 7.34 | 1.05 | 0.29 |
| 3 | 2 | -2.05 | 5.82 | -0.35 | 0.72 |

Section D

| MRA Score | | Mean | SD | Z | p-value |
|---|---|---|---|---|---|
| 1 | 0 | -1.01 | 2.53 | -0.40 | 0.69 |

| | | | | | |
|---|---|---|---|---|---|
| 2 | 0 | 8.47 | 3.83 | 2.21 | 0.03 |
| 2 | 1 | 8.50 | 3.93 | 2.16 | 0.03 |
| 3 | 0 | 7.66 | 6.86 | 1.12 | 0.26 |
| 3 | 1 | 11.14 | 7.34 | 1.52 | 0.13 |
| 3 | 2 | -12.81 | 5.82 | -2.20 | 0.03 |

Section E

| MRA Score | | Mean | SD | Z | p-value |
|---|---|---|---|---|---|
| 1 | 0 | -1.69 | 2.53 | -0.67 | 0.51 |
| 2 | 0 | 6.93 | 3.83 | 1.81 | 0.07 |
| 2 | 1 | 8.00 | 3.93 | 2.04 | 0.04 |
| 3 | 0 | -1.11 | 6.86 | -0.16 | 0.87 |
| 3 | 1 | 3.62 | 7.34 | 0.49 | 0.62 |
| 3 | 2 | -16.06 | 5.82 | -2.76 | 0.006* |

* Statistically significant with Bonferroni correction, p<0.0083

**Supplementary Table 2.** Comparison of enhancement ratio between MRA scores in each section for adult patients.

Section A

| MRA Score | | Mean | SD | Z | p-value |
|---|---|---|---|---|---|
| 1 | 0 | 2.13 | 1.79 | 1.19 | 0.23 |
| 2 | 0 | 2.49 | 2.33 | 1.07 | 0.29 |
| 2 | 1 | -0.89 | 2.40 | -0.37 | 0.71 |
| 3 | 0 | 11.83 | 6.61 | 1.79 | 0.07 |
| 3 | 1 | -0.71 | 8.02 | -0.09 | 0.93 |
| 3 | 2 | 2.82 | 5.56 | 0.51 | 0.61 |

Section B

| MRA Score | | Mean | SD | Z | p-value |
|---|---|---|---|---|---|
| 1 | 0 | 1.07 | 1.79 | 0.60 | 0.55 |
| 2 | 0 | 3.11 | 2.33 | 1.33 | 0.18 |
| 2 | 1 | 3.11 | 2.40 | 1.29 | 0.20 |
| 3 | 0 | 10.05 | 6.61 | 1.52 | 0.13 |
| 3 | 1 | 9.27 | 8.02 | 1.16 | 0.25 |
| 3 | 2 | -2.96 | 5.56 | -0.53 | 0.59 |

Section C

| MRA Score | | Mean | SD | Z | p-value |
|---|---|---|---|---|---|
| 1 | 0 | 0.00 | 1.79 | 0.00 | 1.00 |
| 2 | 0 | 0.62 | 2.33 | 0.27 | 0.79 |
| 2 | 1 | 0.89 | 2.40 | 0.37 | 0.71 |
| 3 | 0 | 2.23 | 6.61 | 0.34 | 0.74 |
| 3 | 1 | 3.21 | 8.02 | 0.40 | 0.69 |
| 3 | 2 | -1.34 | 5.56 | -0.24 | 0.81 |

Section D

| MRA Score | | Mean | SD | Z | p-value |
|---|---|---|---|---|---|
| 1 | 0 | 0.53 | 1.79 | 0.30 | 0.77 |
| 2 | 0 | 3.11 | 2.33 | 1.33 | 0.18 |
| 2 | 1 | 1.33 | 2.40 | 0.55 | 0.58 |

| | | | | | |
|---|---|---|---|---|---|
| 3 | 0 | 8.71 | 6.61 | 1.32 | 0.19 |
| 3 | 1 | 5.71 | 8.02 | 0.71 | 0.48 |
| 3 | 2 | -4.97 | 5.56 | -0.89 | 0.37 |

Section E

| MRA Score | | Mean | SD | Z | p-value |
|---|---|---|---|---|---|
| 1 | 0 | -1.60 | 1.79 | -0.89 | 0.37 |
| 2 | 0 | 0.00 | 2.33 | 0.00 | 1.00 |
| 2 | 1 | 3.11 | 2.40 | 1.29 | 0.20 |
| 3 | 0 | -1.56 | 6.61 | -0.24 | 0.81 |
| 3 | 1 | 10.34 | 8.02 | 1.29 | 0.20 |
| 3 | 2 | -2.69 | 5.56 | -0.48 | 0.63 |

* Statistically significant with Bonferroni correction, $p<0.0083$

**Supplementary Table 3.** Comparison of enhancement ratio between MRA scores in each section for pediatric patients.

Section A

| MRA Score | | Mean | SD | Z | p-value |
|---|---|---|---|---|---|
| 1 | 0 | -0.40 | 1.91 | -0.21 | 0.83 |
| 2 | 0 | -5.12 | 3.18 | -1.61 | 0.11 |
| 2 | 1 | -1.18 | 3.18 | -0.37 | 0.71 |
| 3 | 0 | 0.92 | 3.18 | 0.29 | 0.77 |
| 3 | 1 | 3.02 | 3.18 | 0.95 | 0.34 |
| 3 | 2 | 9.44 | 3.32 | 2.85 | 0.004* |

Section B

| MRA Score | | Mean | SD | Z | p-value |
|---|---|---|---|---|---|
| 1 | 0 | 0.40 | 1.91 | 0.21 | 0.83 |
| 2 | 0 | -0.92 | 3.18 | -0.29 | 0.77 |
| 2 | 1 | -2.23 | 3.18 | -0.70 | 0.48 |
| 3 | 0 | 1.71 | 3.18 | 0.54 | 0.59 |
| 3 | 1 | 0.13 | 3.18 | 0.04 | 0.97 |
| 3 | 2 | 3.56 | 3.32 | 1.07 | 0.28 |

Section C

| MRA Score | | Mean | SD | Z | p-value |
|---|---|---|---|---|---|
| 1 | 0 | 0.80 | 1.91 | 0.42 | 0.68 |
| 2 | 0 | 3.02 | 3.18 | 0.95 | 0.34 |
| 2 | 1 | 4.07 | 3.18 | 1.28 | 0.20 |
| 3 | 0 | 1.18 | 3.18 | 0.37 | 0.71 |
| 3 | 1 | 0.13 | 3.18 | 0.04 | 0.97 |
| 3 | 2 | -3.56 | 3.32 | -1.07 | 0.28 |

Section D

| MRA Score | | Mean | SD | Z | p-value |
|---|---|---|---|---|---|
| 1 | 0 | -2.00 | 1.91 | -1.04 | 0.30 |
| 2 | 0 | 4.59 | 3.18 | 1.45 | 0.15 |
| 2 | 1 | 6.96 | 3.18 | 2.19 | 0.03 |

| | | | | | |
|---|---|---|---|---|---|
| 3 | 0 | -0.92 | 3.18 | -0.29 | 0.77 |
| 3 | 1 | 1.97 | 3.18 | 0.62 | 0.54 |
| 3 | 2 | -6.56 | 3.32 | -1.98 | 0.048 |

Section E

| MRA Score | | Mean | SD | Z | p-value |
|---|---|---|---|---|---|
| 1 | 0 | 0.00 | 1.91 | 0.00 | 1.00 |
| 2 | 0 | 6.96 | 3.18 | 2.19 | 0.03 |
| 2 | 1 | 5.64 | 3.18 | 1.78 | 0.08 |
| 3 | 0 | -0.66 | 3.18 | -0.21 | 0.84 |
| 3 | 1 | -1.97 | 3.18 | -0.62 | 0.54 |
| 3 | 2 | -8.94 | 3.32 | -2.69 | 0.007* |

* Statistically significant with Bonferroni correction, p<0.0083

**Supplementary Table 4.** The relationship between enhancement ratio and history of TIA.

MRA Score 2

| Section | Mean | SD | Z | p-value |
|---|---|---|---|---|
| A | -2.13 | 2.67 | -0.80 | 0.43 |
| B | 2.13 | 2.67 | 0.80 | 0.43 |
| C | 5.13 | 2.67 | 1.92 | 0.05 |
| D | 1.88 | 2.67 | 0.70 | 0.48 |
| E | 6.13 | 2.67 | 2.29 | 0.02* |

MRA Score 3

| Section | Mean | SD | Z | p-value |
|---|---|---|---|---|
| A | -7.59 | 4.48 | -1.69 | 0.09 |
| B | -7.18 | 4.48 | -1.60 | 0.11 |
| C | 3.34 | 4.48 | 0.75 | 0.46 |
| D | 0.10 | 4.48 | 0.02 | 0.98 |
| E | 3.54 | 4.48 | 0.79 | 0.43 |

Note that no TIA event was observed in patients with MRA Score 0 and 1. The asterisk (*) represents statistical significance (p values <0.05). TIA, transient ischemic attack.

## Supplementary Figures

**Supplementary Figure 1.** Case 2. Left-side ICA-MCA of a 7-year-old girl.

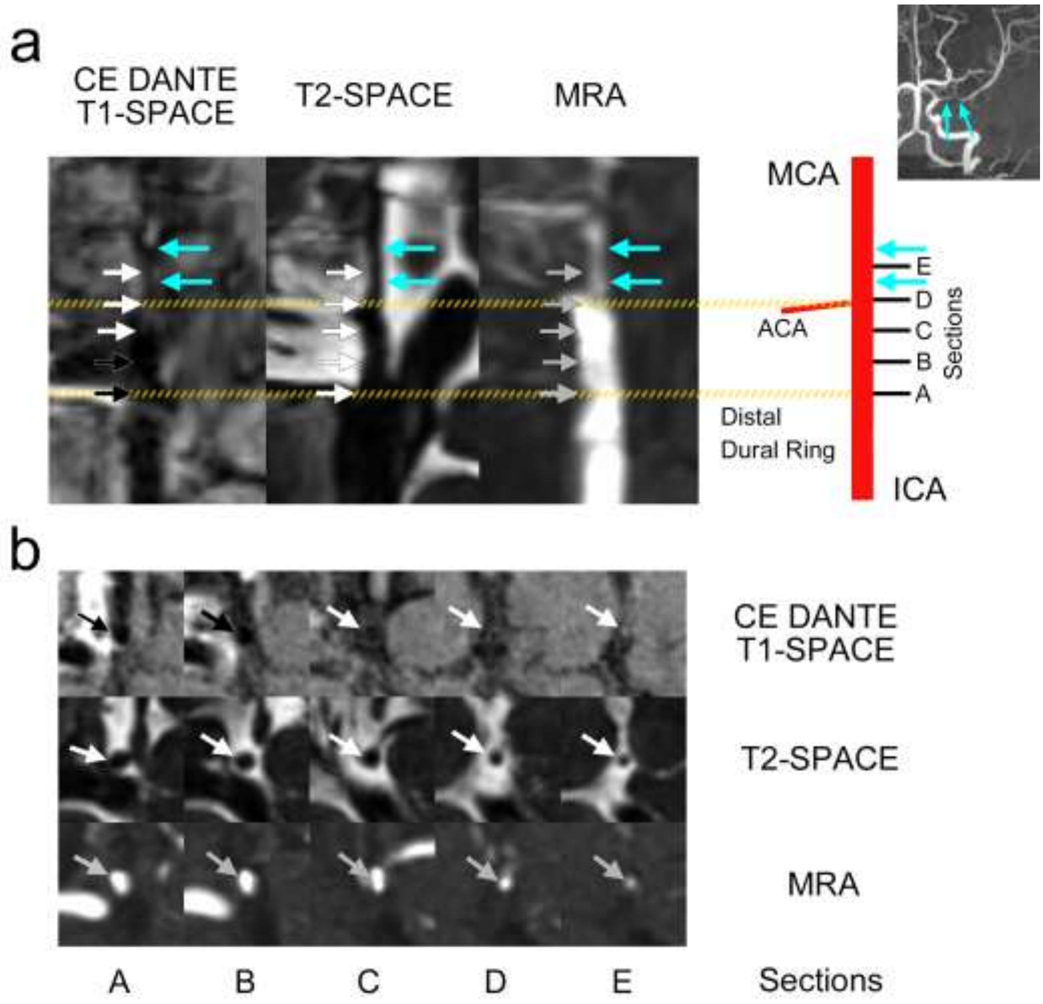


On the MIP image, stenosis of the distal ICA to proximal MCA is evident, but the signal is continuous (MRA score 1, cyan arrows). a) Stretched MPR images of CE DANTE T1-SPACE, T2-SPACE, and MRA and schema of the 5 observed sections. Note that distances between each section are equal. Section A, start of the intracranial ICA (across the distal dural ring); Section B, proximal intracranial ICA; Section C, distal intracranial ICA; Section D, end of the ICA; and Section E, proximal MCA. On MRA, stenosis is seen from the distal ICA to the proximal MCA, but outer diameter is relatively preserved on T2-SPACE (arrows). CE T1-DANTE shows subtle enhancement (arrows). b) Axial stretched MPR images. CE T1-DANTE shows weak contrast enhancement of the arterial wall in Section E (top row). White, black, and gray arrows indicate the ICA-MCA in Sections A–E.

**Supplementary Figure 2.** Case 3. Left-side ICA-MCA of a 47-year-old woman.

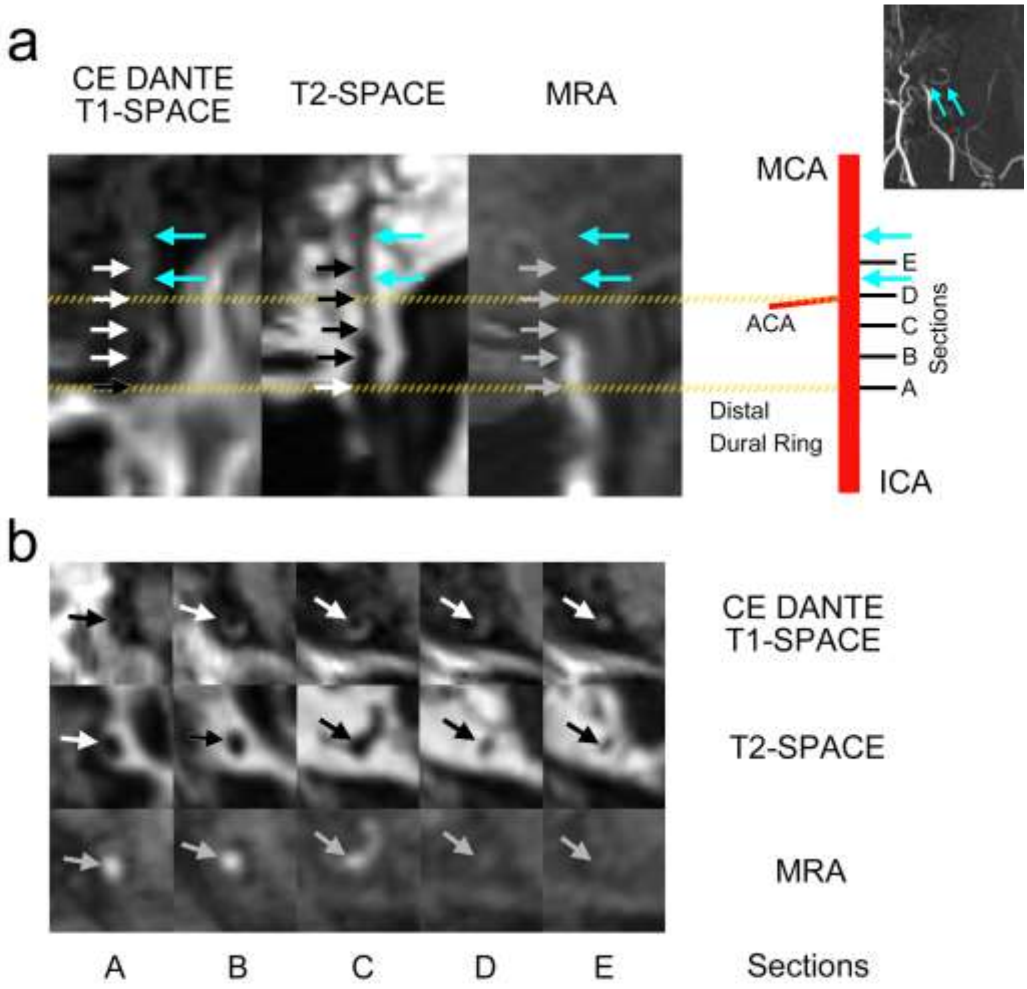


On the MIP image, the distal ICA to proximal MCA is invisible (MRA score 3). a) Stretched MPR images of CE DANTE T1-SPACE, T2-SPACE, and MRA and schema of the 5 observation sections. Note that distances between each section are equal. Section A, start of the intracranial ICA (across the distal dural ring); Section B, proximal intracranial ICA; Section C, distal intracranial ICA; Section D, end of the ICA; and Section E, proximal MCA. On MRA, the distal ICA to proximal MCA is invisible and the artery has shrunk on the T2-SPACE image (arrows). CE T1-DANTE shows weak enhancement (arrows). b) Axial stretched MPR images. CE DANTE T1-SPACE shows weak contrast enhancement on the arterial wall in Sections B–E (top row). In Sections D and E, the artery is invisible on MRA and displays reduced outer diameter. White, black, and gray arrows indicate the ICA-MCA in Sections A–E.

**Supplementary Figure 3.** Case 4. Left-side ICA-MCA of a 50-year-old woman.

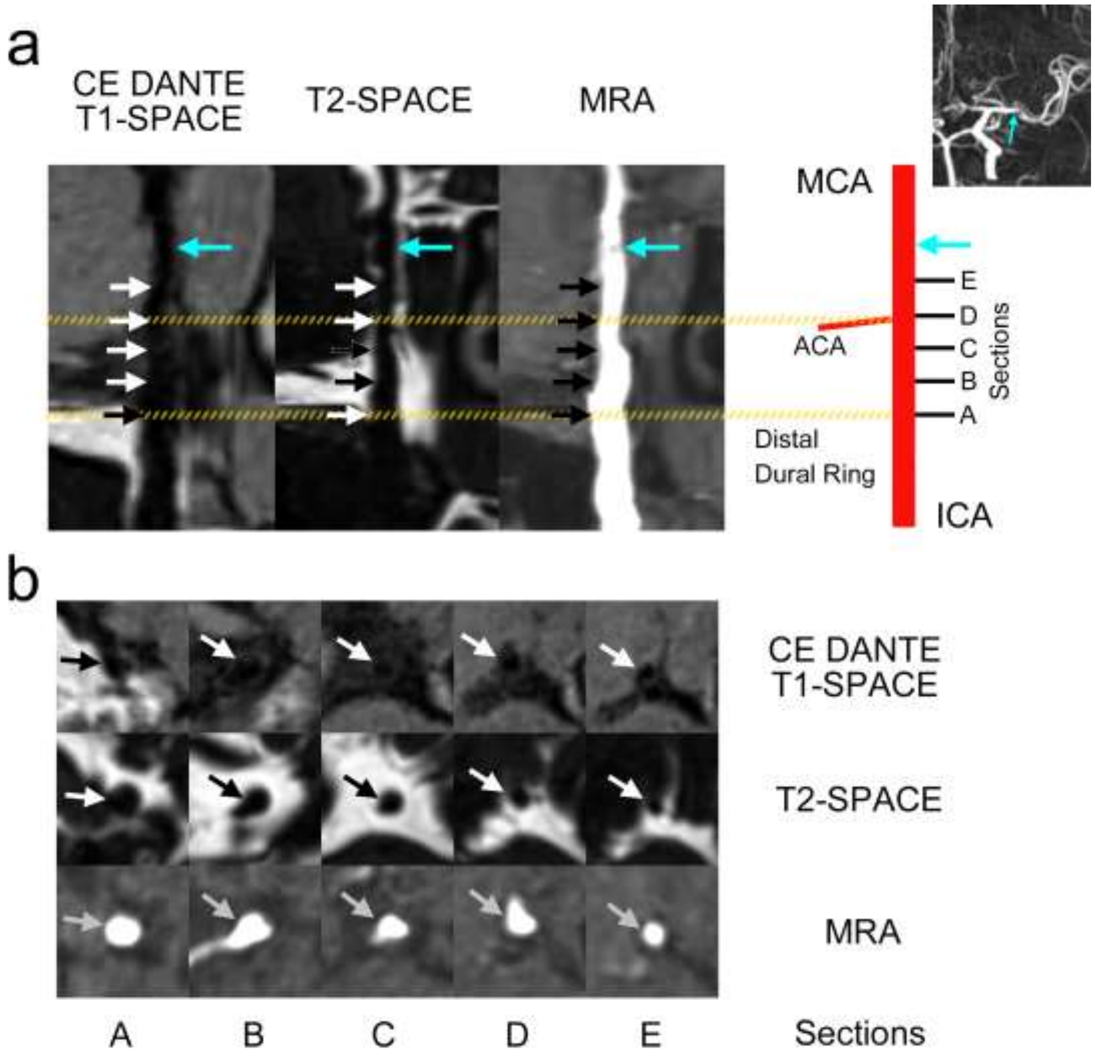


On the MIP image, the distal ICA to proximal MCA appears normal (MRA score 0). Mild stenosis is found in the distal MCA (cyan arrows). a) Stretched MPR images of CE DANTE T1-SPACE, T2-SPACE, and MRA and schema of the 5 observed sections. Note that distances between each section are equal. Section A, start of the intracranial ICA (across the distal dural ring); Section B, proximal intracranial ICA; Section C, distal intracranial ICA; Section D, end of the ICA; and Section E, proximal MCA. The distal ICA to proximal MCA appears normal on MRA and T2-SPACE. CE DANTE T1-SPACE shows no definitive arterial wall enhancement (arrows). b) Axial stretched MPR images. No stenotic or enhanced lesions are evident. Note that white, black, and gray arrows indicate the ICA-MCA in Sections A–E.

**Supplementary Figure 4.** Case 1. ROIs of CE DANTE T1-SPACE and T2-SPACE of the 5 observed sections are shown.

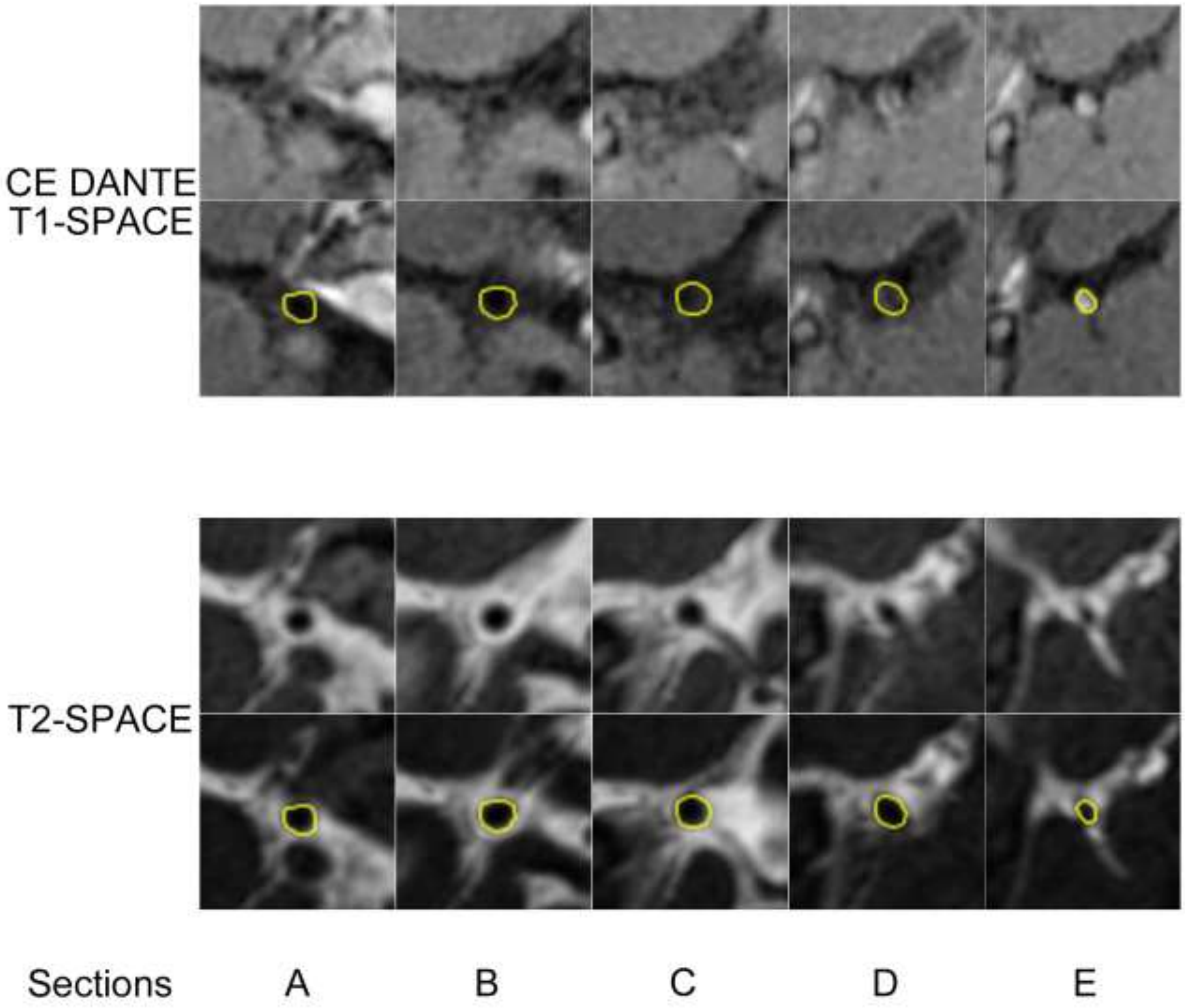


ROI was manually set along the outer contour of the artery including the lumen. CE DANTE T1-SPACE shows enhancement from Section D to E (end of ICA to proximal MCA), T2-SPACE shows mild narrowing of the artery. Section A, start of the intracranial ICA (across the distal dural ring); Section B, proximal intracranial ICA; Section C, distal intracranial ICA; Section D, end of the ICA; and Section E, proximal MCA.
